# Noncompliance with Medical Regimen in Haemodialysis Treatment: A Case Study

**DOI:** 10.1155/2011/476038

**Published:** 2011-12-19

**Authors:** Paraskevi Theofilou

**Affiliations:** Department of Psychology, Panteion University, Eratous 12, 14568, Athens, Greece

## Abstract

Patients undergoing haemodialysis treatment have a high burden of disease (particularly cardiovascular comorbidities) affecting their quality of life and dramatically shortening life expectancy. Effective chronic kidney disease (CKD) control requires regular preventive medication and a response to that medication. Poor receptiveness to CKD medication can be related to individual variability in the dose needed to achieve a response, as well as to low-adherent behaviour in relation to the CKD medication regimen. Some patients, though not many, according to studies' findings, abuse the medical regimen as a result of suicidal tendencies. The present case gave us the opportunity to consider the causes and clinical findings and review the specific psychological interventions for patients with CKD.

## 1. Introduction

In many areas of medicine, the patient's well-being and health greatly depend on the active participation in a treatment program and the compliance with the medical recommendations, for example, taking medicines, adhering to a diet or undergoing surgery. Since abuse of medical recommendations often threatens the patient's health and life, there exists a tendency to regard noncompliance as expressing suicidal intentions (conscious or unconscious) and to regard it as suicidal behaviour [1].

The chronic haemodialysis (HD) setup is ideal for studying the problems of noncompliance and abuse of medical regimen. The treatment is chronic and the contact with the patients both prolonged and intensive.

Patients undergoing HD have to cope with many adversities, for example, physical symptoms, limitations in food and fluid intake, changes in their body image, work and economic status, social roles, activity levels, self-image, health status, and normal routines, while their control over treatment cannot always be predicted [1–3]. Such constraints are expected to affect the patients' life and physical as well as social functioning, leading them to reconsider their personal and professional goals within the context of living with a chronic illness [4–8].

Regarding mental health, much attention has been given to depression in patients on maintenance dialysis during the past three decades. Considered to be the most common psychiatric abnormality in patients with chronic kidney disease (CKD) [9, 10], depression has been studied extensively in these individuals. The effect of depression on mortality, suicide, and hospitalization has been studied recently, and depression is established as an independent risk factor for mortality and morbidity [11–14]. Drayer and colleagues found that patients with CKD and depression have 4.1 times the mortality rate of patients with CKD who do not have depression [15]. Lopes and coworkers suggested a higher rate of withdrawal from maintenance dialysis in patients who developed depression while on dialysis than in those patients who were not depressed. These investigators also found a higher risk of mortality and hospitalization associated with depression [14]. Einwohner and colleagues showed a similar increased risk of morbidity and mortality in depressed patients on maintenance peritoneal dialysis [16]. Suicide is highly linked with a depressed state of mind [17, 18]. It accounts for a death rate of 0.2% per 1000 dialysis patients/years at risk [17, 18]. Kurella and colleagues also found a strong association between alcohol dependence and hospitalization for substance abuse and mental illness with subsequent death by suicide [19]. Patients with CKD are often poorly adherent to their medical regimens, and Cukor and coworkers have reported depression as an independent factor for nonadherence in patients on maintenance dialysis [20].

HD patients have been found to experience depressive symptoms [21, 22]. Depression may be linked to the HD treatment modality, since the patient has to be continually connected to the haemodialysis machine during dialysis and so experience significant restrictions in independent living [21, 22]. In addition, the rate of reported suicide in HD is higher, while a substantial number of deaths resulting from dietary violations could also be accounted for as suicide [23, 24].

This paper presents a case of a male patient with severe depression who received HD treatment.

## 2. Case Report

A Greek man of fifty-six years old, who was diagnosed as having end-stage renal disease at age of 54 on the basis of persistent proteinuria with hematuria, started undergoing HD treatment. He is married and pensioner. When he became accustomed to dialysis treatment, he experienced a honeymoon period during which he perceived his treatment as being far less horrific and unmanageable than anticipated. In other words, he felt appreciation for the improvement in his health brought by dialysis. Further, his openness to transplant was low in this phase of illness adjustment, as he believed that he can remain on dialysis with minimal disruptions to his life. This stage lasted for six months.

Next, he entered a transitional state during which he experienced confusing emotions, disregard medical recommendations and distrust medical professionals. Frustration with the rigor of treatment regimens led to noncompliance with medical tasks and complacency with poor health. Also, he wavered between hopelessness about his medical condition and euphoria at the prospect of a possible transplant or other treatment that could enhance quality of life. This stage lasted for one year.

For about seven months, this patient presents major depressive symptomatology, including symptoms of dysphoric mood, loss of interest or pleasure, sleep disturbance, fatigue, feelings of worthlessness, as well as suicidal ideation. He believes that the desired outcome, which is getting well through transplantation, will not occur and that he is powerless to either prevent negative outcomes or generate positive health outcomes. Further, he does not accept his wife's support and giving help, he forgets taking his medicines systematically and he does not follow doctor's instructions regarding limitations in food and fluid intake. He drinks too much, he eats forbidden foods and often says “I am fed up”.

## 3. Discussion

Patients with CKD face many challenges which increase the likelihood that they will develop depression or anxiety or worsen these conditions. These include a general feeling of unwellness; specific symptoms caused by CKD or the patient's treatment; major disruptions in lifestyle; the need to comply with treatment regimens, like dialysis schedules, diet prescription, and water restriction; ancillary treatments and hospitalizations; the fear of disability, morbidity, and shortened lifespan [25, 26].

Consequently, major depression is the most common psychiatric disorder reported among adults with CKD, including symptoms of dysphoric mood, somatic concerns, difficulty concentrating, loss of interest or pleasure, decreased or increased appetite, weight change, sleep disturbance, psychomotor agitation or retardation, fatigue, aches/pains, guilt, feelings of worthlessness, suicidal ideation, or preoccupation with death [25, 26]. Willingness to participate in transplant education programs may be hindered by suicide thoughts or feelings of worthlessness and guilt, such that patients do not believe they are “worthy” of a transplant or may be excessively worried about the risks to a living donor despite the medical facts regarding favourable outcomes among kidney donors. Health professionals should stress the benefits of transplantation not only for recipients, but for donors as well.

Individuals with depression also have information processing biases, such that they have difficulty inhibiting negative information and retrieve it more efficiently than positive information [27]. In other words, depressed patients think negatively about themselves as well as their past, present, and future experiences. In our case, patient's depression has been associated with learned helplessness [28], a phenomenon in which individuals believe no behavior will achieve their desired outcome [29]. Similarly, a patient may also exhibit learned hopelessness or the belief that desired outcomes (e.g., getting well or getting a transplant) will not occur, and that he or she is powerless to either prevent negative outcomes or generate positive health outcomes [28]. Depression is linked to reduced self-efficacy [30, 31], deficits in planning [32] and contemplating rather than initiating behavior change [33]. Treatment education should foster independence and encourage action, which may reduce the development of learned helplessness among adults with CKD [34], and address the impaired autonomy often perceived by persons with CKD [35].

In patients with CKD, the symptoms of depression or anxiety may be similar to those that occur with kidney failure or uremia per se. A team approach that includes psychologists, psychiatrists, or social workers is generally needed in order to identify, comprehensively diagnose, and treat these illnesses. Pharmacological treatment, psychotherapy, and cognitive behavioral therapy have been the mainstay for the treatment of depression and anxiety in the general population [36, 37]. Similar options are available to treat the psychological disorders in patients with CKD [36]. Cognitive behavioral therapy has been shown to improve symptoms of depression in patients on maintenance dialysis [38, 39]. Exercise programs may also be effective at reducing depressive symptoms in these individuals [40, 41]. Social support is also an integral way of providing better healthcare for patients with CKD. Integration of psychological and material support for better healthcare of the patient is the essence of the social support system. Social support has been shown to have an improved end result in a variety of chronic illnesses [42, 43]. Symister and Friend as well as Theofilou reported that social support decreased depression by improving the self-esteem of the patients with CKD, which led to an increased degree of optimism in these patients [44–47].
